# Health promotion and disease prevention in the education of health professionals: a mapping of European educational programmes from 2019

**DOI:** 10.1186/s12909-022-03826-5

**Published:** 2022-11-11

**Authors:** Kristiina Patja, Tessa Huis in ‘t Veld, Dorottya Arva, Marjorie Bonello, Rana Orhan Pees, Marc Soethout, Martin van der Esch

**Affiliations:** 1 Department of Public Health, Medical Faculty, University of Helsinki, PO BOX 20 (Tukholmankatu 8 B), 00014 Helsinki, Finland; 2 Ecorys Nederland BV, Watermanweg 44, 3067 GG Rotterdam, The Netherlands; 3 Department of Public Health, Faculty of Medicine, Semmelweis University, Nagyvárad tér 4, 1089 Budapest, Hungary; 4 MTA-PTE Innovative Health Pedagogy Research Group, University of Pécs, Pécs, Hungary; 5 Department of Occupational Therapy, Faculty of Health Sciences, University of Malta, Msida, MSD 2090 Malta; 6 European Medical Students’ Association (EMSA), c/o CPME, Rue Guimard 15, 1040 Brussels, Belgium; 7 Association of Schools of Public Health in the European Region (ASPHER), UM Brussels Campus, Av de Tervueren 153, BE-1150 Brussels, Belgium; 8 Department of Public and Occupational Health, Amsterdam University Medical Centre, Van der Boechorststraat 7, 1081 BT Amsterdam, The Netherlands; 9 Centre of Expertise Urban Vitality, Faculty of Health, Amsterdam University of Applied Sciences, Weesperzijde 190, 1097 DZ Amsterdam, The Netherlands; 10 Reade, Center for Rehabilitation and Rheumatology, J. van breemenstraat 2, 1056 AB Amsterdam, The Netherlands

**Keywords:** Non-communicable diseases, Health promotion, Disease prevention, Education, Health professionals, Teaching methods

## Abstract

**Background:**

Health professionals face barriers in carrying out effective health promotion and disease prevention. To indicate what are the needs for curriculum development in educational programmes, this study aims to provide an overview of how various health professionals are currently trained in health promotion and disease prevention at different educational levels.

**Methods:**

In 2019, a descriptive mapping exercise was performed focusing on European programmes for different health and healthcare professionals at the three levels of education (undergraduate, postgraduate, and continuous professional development [CPD]). Data were collected by a self-developed online survey that was distributed using a modified snowball method.

**Results:**

A total of 186 educational programmes of 17 different health professionals were analysed, implemented in 31 countries (60% were undergraduate, 30% postgraduate and 10% CPD programmes). Nearly all programmes indicated that expected outcomes were defined on knowledge (99%), skills (94%) and behaviours/attitudes (89%) regarding health promotion and disease prevention. A multidisciplinary approach was reported to be applied by 81% of the programmes. Traditional teaching methods such as lectures (97%) and assignments (81%) were dominant, while e-learning was less frequently used (46%). Digitalization in health promotion and digital health coaching were the least addressed topics in most programmes.

**Conclusions:**

Health promotion and disease prevention are reported at all surveyed levels of education for a broad spectrum of health professionals. Educational programmes cover contents on knowledge, skills, and behaviours. There is a need for capacity building and joint development in health promotion education. Specifically, there is a need to include digitalisation and novel teaching in the educational programmes of health promotion and disease prevention.

**Supplementary Information:**

The online version contains supplementary material available at 10.1186/s12909-022-03826-5.

## Background

Non-communicable diseases (NCDs) cause nine out of ten deaths in the European region, of which six out of ten can be attributed to common modifiable risk factors such as unhealthy diet, tobacco use, alcohol use, physical inactivity and environmental factors [1]. Strengthening the investments in health promotion and disease prevention by empowering health and healthcare professionals can reduce the burden, as these professionals are uniquely positioned to advocate healthy behaviours [2–4]. Providing high quality education in health promotion at all levels of education from undergraduate to continuous professional development (CPD) represents such an investment [5]. Several identified barriers of healthcare professionals in providing effective counselling on healthy life behaviours could be targeted by training, like lack of competencies and confidence [4], doubts about patients’ acceptance and willingness to receive information on healthy life behaviours [6], insufficient skills and training, misconceptions about the effectiveness of interventions and health promotion perceived as outside of professional role [7–9]. Infrastructural barriers like perceived lack of time or competing workload and insufficient reimbursement need to be acknowledged as well [6]. However, at all levels of education, there seems to be a mismatch between roles, competencies, training and realisation among healthcare professionals and care delivery [7].

With growing burden of NCDs in Europe, education in health promotion and disease prevention is needed in all its countries and at all levels of education: undergraduate, postgraduate, and continuous professional development (CPD) [5, 8]. Before recommendations can be made on how to improve education about the means of promoting healthy behaviours [10], a first step is to make an inventory of current education in these topics. Therefore, this study aimed to provide an overview of how the various health and healthcare professionals are educated in health promotion and disease prevention at different educational levels in the year of 2019, with the focus on European educational programmes.

## Methods

As part of a two-year project (2018–2020) of the European Union (EU) commissioned by Consumers, Health, Agriculture and Food Executive Agency (Chafea) – European Commission a mapping of education of health promotion and disease prevention was carried out at different levels of training programmes for health professionals.

Given that no existing mapping tool was available for this purpose, an online questionnaire was developed in several steps detailed in Additional file 1. The classification of learning outcomes by Kraiger [11] served as theoretical framework of the questionnaire.

### Data collection

Since the aim of the project was to map current educational programmes of health professionals throughout Europe and relevant informants were hard to find via only governmental routes, the snowball methodology was chosen to obtain information. First, an invitation to collaborate and fill in the questionnaire was sent by email to 860 potential informants representing a network of professionals: to 26 major European educational and professional networks, to 252 national health associations and to 584 national educational organisations (see Additional file 1 for further information on the approached informants). All approached informants were asked to forward the questionnaire among their national and/or international colleagues or members to recruit additional informants via their networks.

Although this recruitment method required more effort and time at the beginning, it increased the reliability that relevant informants were found. Once the ‘snowball’ was rolling, it provided us with a growing dataset including the perspectives from health educators, healthcare professional associations, healthcare students and residents. In addition, it served as a communication and dissemination vehicle.

The questionnaire was distributed between September 2019 and February 2020. The questionnaire was open for any training programme of health professionals at either under-, postgraduate or CPD level. One questionnaire represented one educational programme, thus an informant who had information about several educational programmes could fill in multiple questionnaires.

The question *‘Does the education/course cover the topic of health promotion and disease prevention?’* was used as inclusion criteria. All programmes covering these topics (answered ‘Yes’) were included in the overview. Programmes from outside of the World Health Organization (WHO) European Region were excluded.

### Variables

The survey included several topics of which the following variables are presented here (see Additional file 1 for the exact wording of questions):

- level of education *(undergraduate/postgraduate/continuous professional education)*
- accreditation *(on European or international level/on national level/no accreditation/unknown/other)*
- trained health professionals *(medical specialists/general practitioners/medical doctors, non-specialised/physical therapists/occupational therapists/nurses/psychologists/dentists/social workers/other)*
- type of health professionals teaching *(medical doctors/medical specialists/physical therapists/occupational therapists/nurses/psychologists/dentists/social workers/other)*
- approach used *(mono/multidisciplinary/unknown)*
- way of incorporating the topic of health promotion and disease prevention in the curriculum *(one full module is primarily dedicated/topic is covered in all [or most] modules)*
- expected outcomes *(knowledge/skills/behaviour or attitudes)*
- teaching methods *(lectures/assignments/field training in real environments/eLearning modules/other)*
- content of education *(*e.g.*, ethics/health inequalities/health behaviour change techniques)*.

### Data analysis

Data were analysed using IBM SPSS version 28. Variables and their frequencies by level of education were analysed descriptively.

## Results

In total, information was obtained on 208 educational programmes of which 186 covered the topic of health promotion and disease prevention. All programmes were from countries of the WHO European Region, resulting in a final sample of 186 programmes implemented in 31 different countries (for a detailed list of countries see Additional file 2). It is not possible to provide the exact number of informants who received the survey with the snowball method.

The educational programmes represented different levels of education (data were available for 178 out of 186 programmes): 60% (*n* = 107) were undergraduate, 30% (*n* = 53) postgraduate and 10% (*n* = 18) CPD programmes. 40% (*n* = 74) of educational programmes were accredited on European or international level, 52% (*n* = 96) were accredited on national level, and only 4% (*n* = 7) lacked accreditation. The educational programmes targeted 17 different health professionals (see Table 1). From the 186 programmes, 18% (*n* = 34) targeted trainees from multiple professions in one programme (multidisciplinary programmes). When looking at the different educational levels, 11% of undergraduate (*n* = 12), 9% of postgraduate programmes (*n* = 10) and 44% of CPD courses (*n* = 8) were multidisciplinary programmes. Table 1 presents the distribution of programmes between each examined level of education by the targeted health professionals.

**Table 1:** The distribution of programmes between each examined level of education by the health professionals trained

Health Professionals Trained	Number of programmes					
	Undergraduate		Postgraduate		CPD	
Total of health professionals trained in educational programmes^**a**^	100%	167	100%	106	100%	37
Dentists	5%	9	3%	3	8%	3
Dietitians	10%	17	4%	4	5%	2
Kinesiologists	1%	2	–	–	–	–
Lifestyle coaches	1%	1	–	–	–	–
Medical doctors	19%	32	46%	49	25%	9
Midwifes	2%	3	2%	2	–	–
Nurses	11%	18	12%	13	16%	6
Nutritionists	3%	5	2%	2	–	–
Occupational therapists	8%	13	8%	9	8%	3
Osteopathists	–	–	1%	1	–	–
Pharmacists	7%	11	3%	3	–	–
Physical therapists	27%	46	15%	16	11%	4
Public health scientist	1%	2	–	–	–	–
Psychologists	2%	4	1%	1	16%	6
School teachers	–	–	–	–	3%	1
Social workers	2%	3	3%	3	8%	3
Speech therapists	1%	1	–	–	–	–

^a^The total number of programmes listed by trained health professionals is higher than the total number of educational programmes due to multidisciplinary programmes, in which multiple health professionals were trained in one programme. Answers given in categories of ‘medical specialists’, ‘general practitioners’ and ‘medical doctors, non-specialised’ were collapsed under the category of medical doctors, while some professions in the table appeared under the category of ‘other’

Regarding the teachers’ profession, some disciplines (e.g. medical specialists, nurses, and psychologists) were represented more often as an educator than as a trainee. This implies that the educators were involved in programmes oriented towards other disciplines (multidisciplinary approach). In line with this, a multidisciplinary approach was reported to be applied by 81% (*n* = 120) of the programmes (from 148 out of 186).

For 147 out of 186 educational programmes it was indicated if their programme incorporated health promotion and disease prevention in all or most modules of the curriculum (63%, *n* = 93) or in a separate module primarily dedicated to these topics (37%, *n* = 54). Looking at the different levels of education, a similar pattern was found at under- and postgraduate levels, but among CPD programmes, the proportion of modules primarily dedicated to health promotion was more restricted (15%).

For 142 out of 186 educational programmes the expected outcome of education was stated. Nearly all programmes indicated knowledge (99%, *n* = 140), skills (94%, *n* = 134) and behaviours/attitudes (89%, *n* = 127) as the expected outcome of their educational programme, with similar proportions at each examined level of education.

**Fig. 1:**
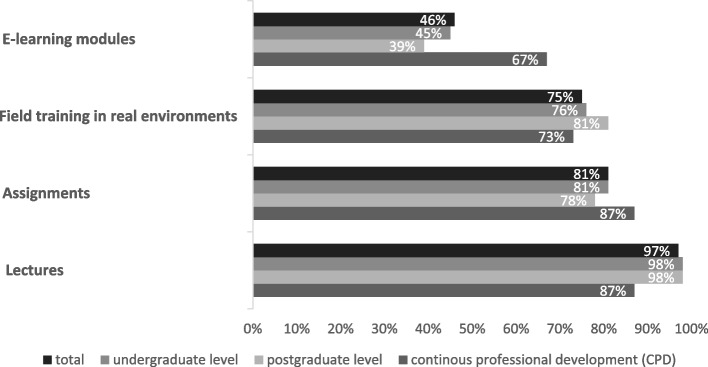
Teaching methods applied in educational programmes by the level of education (*n* = 145)

For 145 out of 186 educational programmes the different teaching methods applied for the topics of health promotion and disease prevention were described. Out of these, most educational programmes (95%) made use of multiple teaching methods. Traditional teaching methods, such as lectures (97%, n = 140) and assignments (81%, *n* = 117) were dominant. While other teaching methods such as field training (75%, *n* = 108) and e-learning methods were used less often (46%, *n* = 67). In CPD, e-learning methods were more often used (67%) compared to under- (45%), and postgraduate education (39%). The proportion of field training was relatively equal at all levels (Fig. 1).

**Fig. 2:**
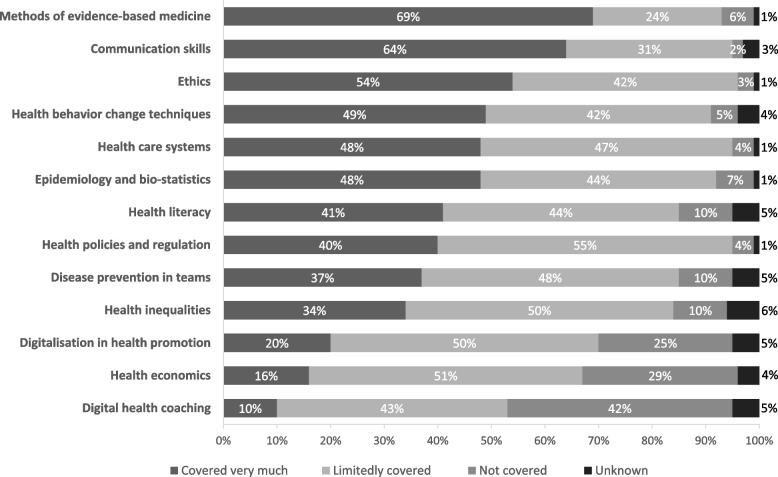
Contents covered in the educational programmes (*n* = 137)

For 137 out of 186 educational programmes the contents covered were described in more detail (Fig. 2). It was found that contents related to attitudes, such as evidence-based medicine (69%, *n* = 95) and ethics (54%, *n* = 74) were covered to a large extent in most educational programmes. Contents which focus on skills such as communication skills (64%, *n* = 87) and health behaviour change techniques (49%, *n* = 67) were covered in depth in around half of all educational programmes. Less attention was given to contents related to skills required in a digital environment, such as digital health coaching (included very much in 10%, *n* = 13) and digitalisation in health promotion (20%, *n* = 28). Digital health coaching and digitalisation in health promotion were more commonly covered in depth in CPD programmes (27% [*n* = 4]; 40% [*n* = 13]) than among undergraduate (5% [*n* = 4]; 17% [*n* = 6]), or postgraduate programmes (13% [*n* = 5]; 25% [*n* = 10]). In addition, it is noteworthy that knowledge on health inequalities and health literacy (covered very much in 34% [*n* = 47] and 41% [*n* = 56] respectively) were not at all included in 10% of educational programmes, while social inequalities are a major risk factor for poor health.

## Discussion

To our knowledge, this is the first cross mapping study focusing on Europe which shows how the topics of health promotion and disease prevention are addressed in educational programmes among different health and healthcare professions. Information was obtained on 186 educational programmes, targeting 17 different health professions. Health promotion and disease prevention were incorporated at all three surveyed levels of education (under-, postgraduate education and CPD) for a wide spectrum of professionals. Accreditation for these educational programmes is common and the use of a multidisciplinary approach seems widespread. Some of the programmes were also targeted at multiple health professionals. Traditional teaching methods, such as lectures (97%) and assignments (81%) were dominant, while e-learning was less frequently used (46%). Epidemiology, evidence-based medicine, health policies and communication skills were most covered and digitalization in health promotion, digital health coaching, health economics and health inequalities were covered the least frequently.

In addition, the results indicate that educational programmes aim to address knowledge, skills and behaviours that may limit health professionals’ activity in effective health promotion and disease prevention on both institutional and interpersonal levels found in previous studies [7, 8]. This is in contrast with earlier findings from the United States, where current education of healthcare professionals seems to have a dearth of health promotion and disease prevention emphasis [6].

Whilst this is encouraging and bears a positive message regarding the growing burden of NCDs, the results also point out future challenges for both educational and health policy development. The environment for healthcare and education is in turmoil as new health threats, like the climate crisis and the current COVID-19-pandemic will have an impact on (digital) competencies needed for professionals [12]. Moreover, the digitalisation of services challenges healthcare professionals [13]. This paradigm shift requires new skills and attitudes from health professionals. Skills are developed through practice and active participation, while multi-method pedagogy enhances skill building [14], so it is worrisome that only half of programmes reported skill building in health behaviour change techniques and only 15% in digital environments. Understanding of factors influencing abilities of individuals and their social environment in adapting to change affect the attitudes of professionals seen as barrier for patient compliance in lifestyle changes and treatments [15]. Therefore, educational programmes need to pay more attention to health literacy, digital health coaching and digitalisation in health promotion and disease prevention, to equip professionals with skills and attitudes to fully benefit patients’ care and abilities to implement them. Additionally, it requires an investment from educational programmes to apply novel teaching techniques including e-learning modules above traditional methods which was found most common in this study.

We believe that to increase the amount of health promotion and disease prevention in healthcare systems, common healthcare professional competencies and context-based implementation strategies are needed across the EU and Europe. A similar need has been recognised in the United States [16–18] and in previous studies in Europe [9, 13]. Our results could be used as a starting point to develop a common framework for implementing competencies for health promotion and disease prevention by health professions in the EU, supplementing the recently updated [19]. Such an initiative might be timely amongst other challenges like changing demographics, digitalisation, and the current COVID-19 pandemic, which has been revising and extending the roles of professionals in healthcare and health promotion [20, 21]. However, the existing multidisciplinary approach can foster the developmental process, and the EU provides a context for collaboration and dissemination of new standards through accreditation systems: universities and other institutions have already collaborated in the coordination of education e.g. the Bologna Process, for undergraduate education [22] and the EU’s 2005 Directive on the recognition of professional qualifications in CDP [23], and in the establishment of frameworks of competencies (examples include the PHARMINE for pharmacists [24] and MEDINE for medical doctors [25]). Equally, lifestyle medicine is expanding [26–28]. To support people with NCDs by promoting healthy behaviours, common competencies could set up a foundation for outcome-based education [29], reduce barriers between different professions, and promote health professional collaboration [30].

### Limitations of the study

Our study has several limitations. It provides only a snapshot of the current situation, as change over time is likely, especially considering the current COVID-19 pandemic moving teaching online. As no single or defined source of information was available for all levels of education (as e.g. curriculum mapping could have been used only for undergraduate education), the snowball method was used. It is a non-random sampling method, which limits the generalisability of results. Counting only educational programs that covered the topics of health promotion and disease prevention increased the possibility of systematic bias. Therefore, this study cannot provide a comprehensive picture of the proportion of health promotion and disease prevention in the education of health professionals in Europe. This shortcoming implies a risk of selection bias, as educational institutions that have implemented health promotion within their programmes, i.e. the front-runners may have been more inclined to respond than those who are less active. Lastly, the data were self-reported by means of a survey and there may be an information bias, as anyone could have filled in the survey in the name of the programme and respondents could be more positive about their own programmes.

## Conclusions

Health promotion education must adapt new contents and methods faster than ever. Education needs to respond to a paradigm shift from treating to coaching patients, often with digital tools, but equally responding to new needs and expectations from the populations. We believe that to increase the amount of health promotion and disease prevention in healthcare systems in Europe, common competencies of healthcare professionals and context-based implementation strategies are needed across the EU and Europe.

## Supplementary Information

**Additional file 1.** Detailed version of Methods section (list of the approached informants and the questionnaire included). This document contains a detailed description of the methods used for this mapping alongside with the list of European Associations, examples of the national associations and numbers of national educational organisations approached as informants and the questionnaire developed for the mapping.

**Additional file 2.** List of countries. This document contains the list of the 31 WHO European Region countries from which educational programmes were included in the mapping.

## Data Availability

The data that support the findings of this study are available from ECORYS Nederland B.V. but restrictions apply to the availability of these data, which were used under license for the current study, and so are not publicly available. Data are however available from the authors upon reasonable request and with permission of ECORYS Nederland B.V.

## Abbreviations

Chafea: Consumers, Health, Agriculture and Food Executive Agency
COVID-19: Coronavirus Disease 2019
CPD: continuous professional development
EFN: European Federation of Nurses Associations
EU: European Union
MEDINE: Thematic Network on Medical Education in Europe
NCDs: non-communicable diseases
PHARMINE: The Pharmacy Education in Europe project
UEMS: European Union of Medical Specialists
WHO: World Health Organization
WHO-ASPHER: Association of Schools of Public Health in the European Region
WFOT: Federation of Occupational Therapists
