# Supplementary material for: Health promotion and disease prevention in the education of health professionals: a mapping of European educational programmes from 2019
Source: BMC Med Educ. 2022 Nov 11;22:778. doi: 10.1186/s12909-022-03826-5 (PMC9652036; doi:10.1186/s12909-022-03826-5)
Supplement: Supplementary file 1 — Additional file 1. Detailed version of Methods section (list of the approached informants and the questionnaire included). This document contains a detailed description of the methods used for this mapping alongside with the list of European Associations, examples of the national associations and numbers of national educational organisations approached as informants and the questionnaire developed for the mapping. [file 12909_2022_3826_MOESM1_ESM.pdf]

Health promotion and disease prevention in the education of health professionals: a mapping of European educational programmes from 2019

Detailed methods, List of organisations approached by email and the questionnaire

Detailed methods

As part of a two-year project (2018-2020) of the European Union (EU) commissioned by Consumers, Health, Agriculture and Food Executive Agency (Chafea) – European Commission, an international preparatory project group was composed for the mapping of education of health promotion and disease prevention at different levels of training programmes for health professionals. This was a multidisciplinary expert group including a medical specialist in public health, a social psychologist and a physiotherapist. Competency profiles for professions were collected during the project both from Europe as well as internationally (1–9). This served as background for data collection.

Given that no existing mapping tool was available for this purpose, an online questionnaire was developed using the classification of learning outcomes by Kraiger (10) as theoretical framework. In Kraiger's classification scheme three types of outcomes are distinguished: 1) cognitive outcomes or knowledge referring to cognitive or mental competencies, 2) skill-based outcomes or competencies in performing activities and 3) affective outcomes or attitudes referring to competencies related to beliefs or values (10). This framework was chosen in accordance with the identified barriers perceived by healthcare professionals in providing effective health promotion and disease prevention on an interpersonal level (11).

The first version of the questionnaire was tested using a Delphi method by the international project group. The revised version was then piloted and reviewed for accuracy among individual members of the group. The necessity of using different wording for different level programmes became evident, while the core of the questionnaire could remain identical for all levels. The final version of the questionnaire was created on a cloud-based survey tool (CheckMarket). This tool enabled conditional stepwise settings, resulting in common (asked from all respondents with the same wording) and

targeted questions (asked or asked differently depending on the educational level). Both open-ended, single, and multiple-choice questions were used in the questionnaire consisting of a maximum of 35 questions in total (the whole questionnaire is presented in this document below methods).

Data collection

Since the aim of the project was to map current educational programmes of health professionals' throughout Europe, first, informants of different organisations of each country (e.g., universities, professional organisations) providing health promotion and disease prevention education, were contacted. Unfortunately, no informant could have been found to provide a full overview of educational programmes addressing health promotion and disease prevention in their country. Since potential informants were hard to find only via governmental routes, the snowball methodology was chosen to obtain information from different professions and reach a variety of programmes from multiple countries.

Mapping consisted of two rounds: first, an invitation to collaborate and fill in the questionnaire was sent by email to 860 potential informants: to 26 major European educational and professional networks (e.g., European Association of Faculties of Pharmacy [EAFP] or European Union of Medical Specialists [UEMS] etc.), to 252 national health associations (selected from the member lists of the 24 European professional networks e.g., Czech Medical Chamber [CMC], Swedish Association of Physiotherapists [Saco], Association of Bulgarian Ergo Therapists [ABET] etc.) and to 584 national educational organisations (selected from the member lists of the national associations e.g., medical schools, schools of dentists, schools of dieticians etc., see further information on approached actors below methods in this document). Then, as a second round of mapping, all informants were asked to forward the questionnaire among their national and/or international colleagues or members to recruit additional informants via their networks. Although this recruitment method required more effort and time at the beginning, it increased the reliability that relevant actors were found. Once the 'snowball' was rolling, it provided us with a growing dataset including the perspectives from health educators,

healthcare professional associations, healthcare students and residents. In addition, it served as a communication and dissemination vehicle and increased engagement in mapping.

The questionnaire was distributed between September 2019 and February 2020. The questionnaire was open for any training programme of health professionals at either under-, postgraduate or CPD level. One questionnaire represented one educational programme, thus an informant who had information about several educational programmes could fill in multiple questionnaires. In February 2020, approached organisations were invited to a workshop in Luxembourg to elaborate the preliminary results of the survey. As they expressed willingness to further disseminate the questionnaire to other informants within their country and their organisation, members and collaborators, the data collection was kept open until the end of February 2020.

The question *'Does the education/course cover the topic of health promotion and disease prevention?'* was used as inclusion criteria. All programmes covering these topics (answered 'Yes') were included in the overview. Programmes from outside of the World Health Organization (WHO) European Region were excluded.

Variables

The survey included several topics of which the following variables are presented here (see the exact wording of questions below methods):

- level of education *(undergraduate/postgraduate/continuous professional education)*
- accreditation *(on European or international level/on national level/no accreditation/unknown/other)*
- trained health professionals *(medical specialists/general practitioners/medical doctors, non-specialised/physical therapists/occupational therapists/nurses/psychologists/dentists/social workers/other)*

- type of health professionals teaching *(medical doctors/medical specialists/physical therapists/occupational therapists/nurses/psychologists/dentists/social workers/other)*
- approach used *(mono/multidisciplinary/unknown)*
- way of incorporating the topic of health promotion and disease prevention in the curriculum *(one full module is primarily dedicated/topic is covered in all [or most] modules)*
- expected outcomes *(knowledge/skills/behaviour or attitudes)*
- teaching methods *(lectures/assignments/field training in real environments/eLearning modules/other)*
- content of education *(e.g., ethics/health inequalities/health behaviour change techniques)*.

Data analysis

Data were analysed using IBM SPSS version 28. Variables and their frequencies by level of education were analysed descriptively.

References

1. IUPHE. Core competencies and professional standards for health promotion. 2016. http://www.ukphr.org/wp-content/uploads/2017/02/Core_Competencies_Standards_linkE.pdf. Accessed 08 Feb 2021.
2. Frank JR, Danoff D. The CanMEDS initiative: Implementing an outcomes-based framework of physician competencies. Med Teach. 2007; doi:10.1080/01421590701746983.
3. World Health Organization. Transforming and Scaling Up Health Professionals' Education and Training: World Health Organization Guidelines 2013. 2013. https://www.who.int/publications/i/item/transforming-and-scaling-up-health-professionals%E2%80%99-education-and-training. Accessed 07 Feb 2021.
4. Accreditation Council for Graduate Medical Education (ACGME) core competencies. https://www.ecfmg.org/echo/acgme-core-competencies.html. Accessed 6 Feb 2018.

5. WFME/AMSE International Task Force. WFME Global Standards for Quality Improvement in Medical Education: European Specifications For Basic and Postgraduate Medical Education and Continuing Professionel Development. 2007. https://wfme.org/download/the-thematic-network-on-medical-education-in-europe-wfme-global-standards-for-quality-improvement-in-medical-education/. Accessed 07 Feb 2021.
6. Frankel RM, Eddins-Folensbee F, Inui TS. Crossing the patient-centered divide: transforming health care quality through enhanced faculty development. Acad Med. 2011; doi:10.1097/ACM.0b013e31820e7e6e.
7. European Accreditation Council for Continuing Medical Education (EACCME). EACCME criteria for the Accreditation of Live Educational Event. 2016. https://eaccme.uems.eu/accreditationlee.aspx. Accessed 07 Feb 2021.
8. European Parliament and Council of the EU. Directive 2005/36/EC on the recognition of professional qualifications and Regulation. 2013. https://eur-lex.europa.eu/legal-content/EN/ALL/?uri=celex%3A32013L0055. Accessed 08 Feb 2021.
9. Atkinson J, Rombaut B. The 2011 PHARMINE report on pharmacy and pharmacy education in the European Union. Pharm Pract (Granada). 2011; doi:10.4321/s1886-36552011000400001.
10. Kraiger K, Ford JK, Salas E. Application of Cognitive, Skill-Based, and Affective Theories of Learning Outcomes to New Methods of Training Evaluation. J Appl Psychol. 1993; doi:10.1037//0021-9010.78.2.311.
11. World Health Organization Regional Office for Europe. Promoting physical activity in the health sector. Current status and success stories from the European Union Member States of the WHO European Region. 2018. https://www.euro.who.int/__data/assets/pdf_file/0008/382337/fs-health-eng.pdf. Accessed 08 Feb 2021.

List of organisations approached by email

1. ***Approached European Associations*** *(in alphabetic order)*

No.	European Associations
1.	Association for Dental Education in Europe (ADEE)
2.	Association for Medical Education in Europe (AMEE)
3.	Council of European Dentists (CED)
4.	Council of Occupational Therapists for the European Countries (COTEC)
5.	Europe Region World Physiotherapy (ER-WPT)
6.	European Association of Faculties of Pharmacy (EAFP)
7.	European Association of Schools of Social Work (EASSW)
8.	European Federation of Nurse Educators (FINE)
9.	European Federation of Nurses Associations (EFN)
10.	European Federation of Psychologists' Associations (EFPA)
11.	European Medical Association (EMANET)
12.	European Medical Students' Association (EMSA)
13.	European Network of Medical Residents in Public Health (EuroNet MRPH)
14.	European Network of Occupational Therapy in Higher Education (ENOTHE)
15.	European Network of Physiotherapy in Higher Education (ENPHE)
16.	European Pharmacists Forum (EPF)
17.	European Society of Preventive Medicine (ESPREVMED)
18.	European Union of General Practitioners (UEMO)
19.	European Union of Medical Specialists (UEMS)
20.	European Forum of Medical Associations (EFMA)
21.	International Federation of Social Workers Europe (IFSW EUROPE)
22.	Pharmaceutical Group of the European Union (PGEU)
23.	Standing Committee of European Doctors (CPME)
24.	The Association of Medical Schools in Europe (AMSE)
25.	The European Federation of the Associations of Dietitians (EFAD)
26.	The European Lifestyle Medicine Organization (ELMO)

2. ***Examples of the 252 approached national associations with the specification of the approached European Association of which they had membership in.*** One example is given out of the 28 approached associations (one from each EU member states in 2019)***.***

**Profession**	**European Association**	**Examples of approached national associations**
Medical doctors	Standing Committee of European Doctors (CPME)	Czech Medical Chamber (CMC)
Physiotherapists	Europe Region World Physiotherapy (ER-WPT)	Swedish Association of Physiotherapists (Saco)
Social workers	International Federation of Social Workers Europe Region (IFSW EUROPE)	German Professional Association for Social Work (DBSH)
Psychologists	European Federation of Psychologists' Associations (EFPA)	Croatian Psychological Association (HPD)
Occupational therapists	Council of Occupational Therapists for the European Countries (COTEC)	Association of Bulgarian Ergotherapists (ABET)
Dietitians	The European Federation of the Associations of Dietitians (EFAD)	French Association of Nutritionist Dietitians (AFDN)
Dentists	Council of European Dentists (CED)	Irish Dental Association (IDA)
Nurses	European Federation of Nurses Associations (EFN)	Polish Nurses' Association (PTP)
Pharmacists	Pharmaceutical Group of the European Union (PGEU)	Malta Chamber of Pharmacists (MCOP)

3. Number of national educational organisations approached

**Profession**	**Number of approached national educational organisations**
Medical schools	298
Schools of physiotherapy	29
Schools of social work	31
Schools of dietitians	40
Schools of occupational therapists	45
Schools of dentists	85
Schools of pharmacists	56

The questionnaire

Health promotion and disease prevention in educational settings

The European Union has commissioned Ecorys, an international research and consultancy agency, to conduct a project on health promotion and disease prevention in health and educational settings. In order to provide an EU wide overview and to better support national governments, we would like to ask for your cooperation. The project will develop an EU wide overview of the education/training programs for basic education, postgraduate education, as well as continuous professional development with regard to education on health promotion. Your help will therefore be key in the collection of information and data on public health initiatives in medical and non-medical education (including medical doctors, physicians, medical specialists, physiotherapists, nurses, occupational therapists, psychologists, social workers, dentists, pharmacists etc.). The survey will take approximately 20-30 minutes of your time. You do not need to complete this survey at once. You may leave the survey and continue at a later moment in time. Questions can be saved by clicking on 'pause' (below the questionnaire). When doing so, a new URL link will be provided. In case your educational institute also provides postgraduate education for other health professionals such as pharmacists or dentists, etc. we would kindly ask you to forward this email to the educational coordinators of these programs. (THIS SENTENCE WAS INCLUDED ONLY FOR POSTGRADUATE PROGRAMMES) **We would be pleased if you could provide this information by means of an online questionnaire to Ecorys, who is also available in case you have questions or you would like additional information:** CONTACT INFORMATION WAS GIVEN HERE

*
1. What is the name of your education programme?

[ ]

*
2. What is the name of your education institution?

[ ]

*
3. What type of health professionals are trained in the education?*please select all answers that apply

- ○ Medical specialists
- ○ General practitioners
- ○ Medical doctors, non specialized
- ○ Physical therapists
- ○ Occupational therapists
- ○ Nurses
- ○ Psychologists
- ○ Dentists
- ○ Social workers
- ○ Other, please specify ....

Q. No. 4 ONLY APPEARS FOR THOSE WHO HAVE SELECTED “MEDICAL SPECIALISTS” FOR Q. No. 3.

*
4. What type of medical specialist?

*
5. Is the education...

- ○ Undergraduate education
- ○ Postgraduate education
- ○ Continuous professional education

IN Qs. BETWEEN Q. No. 6-7 THE WORDING VARIES DEPENDING ON THE LEVEL OF EDUCATION (MEANING THE ANSWER GIVEN FOR Q. No. 5).

*

6. Is the

(IF UNDER/POSTGRADUATE EDUCATION) **education accreditated?**

(IF CONTINUOUS PROFESSIONAL EDUCATION) **course accreditated?**

- o Yes, accreditated on European/ international level
- o Yes, accreditated on national level
- o No
- o Unknown
- o Other, please specify ....

**

7. Does the

(IF UNDER/POSTGRADUATE EDUCATION) **education**

(IF CONTINUOUS PROFESSIONAL EDUCATION) **course**

cover the topic health promotion and disease prevention?

- o Yes
- o No

*

8. How is the topic health promotion and disease prevention incorporated in the curriculum of the educational programme?

- o One full module is primarily dedicated to health promotion and disease prevention
- o Topic is covered in all (or most) modules of the educational programme

THE WORDING IN SOME Qs. BETWEEN No. 9-15 IS THE SAME FOR UNDER/POSTGRADUATE EDUCATION BUT VARIES DEPENDING IF “ONE FULL MODULE IS DEDICATED”/ “COVERED IN ALL MODULES” WAS SELECTED FOR Q. No. 8, AND IS DIFFERENT ALSO FOR CONTINUOUS PROFESSIONAL EDUCATION.

9. CONTACT PERSONS

(IF ONE FULL MODULE IS DEDICATED) **Who is (are) the contact person(s) of the module health promotion and disease prevention? (if any) Enter the name(s) and mailaddres(ses) of the contact person(s) who has/have the authority to require consultation**

(IF COVERED IN ALL MODULES) **Is there a contact person(s) /expert for the topic health promotion and disease prevention? (if any)**

(IF CONTINUOUS PROFESSIONAL EDUCATION) **Who is (are) the contact person(s) of the course on health promotion and disease prevention? Enter the name(s) and mailaddres(ses) of the contact person(s) who has/have the authority to require consultation**

10. Were there any partnerships in

(IF ONE FULL MODULE IS DEDICATED) **setting up the module health promotion and disease prevention? For example other universities**

(IF COVERED IN ALL MODULES) **incorporating it in the curriculum of the whole educational programme?**

(IF CONTINUOUS PROFESSIONAL EDUCATION) **setting up the course on health promotion and disease prevention? For example other universities**

- o Yes
- o No
- o Unknown

11. Are there any partnerships with regard to the execution of the teaching activities on health promotion and disease prevention?If there are any partnerships (e.g. businesses, NGOs, individual citizens, governmental agencies who have a role) fill in yes, and replace namely (..) by the partnership in place.

- o Yes
- o No
- o Unknown

12. Has the education obtained any funding to

(IF ONE FULL MODULE IS DEDICATED) **set up the module on health promotion and disease prevention?**

(IF COVERED IN ALL MODULES) **incorporate health promotion and disease prevention into the curriculum?**

(IF CONTINUOUS PROFESSIONAL EDUCATION) **set up the course on health promotion and disease prevention?**

EU funding, national public funding, local public funding or commercial private funding (e.g. pharmaceutical companies)

- Yes
- No
- Unknown

*

13. Which health professionals are teaching health promotion and disease prevention?

- Medical doctors
- Medical specialists
- Physical therapists
- Occupational therapists
- Nurses
- Psychologists
- Dentists
- Social workers
- Other, please specify ....

14. Since when is

(IF ONE FULL MODULE IS DEDICATED) **the module on health promotion and disease prevention provided?**

(IF COVERED IN ALL MODULES) **health promotion and disease prevention incorporated into the whole curriculum?**

(IF CONTINUOUS PROFESSIONAL EDUCATION) **the course on health promotion and disease prevention provided?**

e.g. since September 2017

15. What is the typical quantity in hours

(IF ONE FULL MODULE IS DEDICATED) **spend per module (approximately) at health promotion and disease prevention by participants?**

(IF COVERED IN ALL MODULES) **per month (approximately) spend at health promotion and disease prevention by participants?**

(IF CONTINUOUS PROFESSIONAL EDUCATION) **spend per month (approximately) at health promotion and disease prevention by participants?**

The amount of hours spent on attendance and homework

Qs. No. 16-20 APPEAR ONLY FOR UNDER/POSTGRADUATE EDUCATION IF "ONE FULL MODULE IS DEDICATED" WAS SELECTED FOR Q. No. 8 AND FOR CONTINUOUS PROFESSIONAL EDUCATION.

16. What is the timescale of the

(IF ONE FULL MODULE IS DEDICATED) **module?**

(IF CONTINUOUS PROFESSIONAL EDUCATION) **course?**

e.g. is it a two weeks, one month or one year programme?

17. Is the

(IF ONE FULL MODULE IS DEDICATED) **module**

(IF CONTINUOUS PROFESSIONAL EDUCATION) **course**

on health promotion and disease prevention accredited separately?

- o No
- o Unknown
- o Yes, please specify how many points are given ....

18. Is it mandatory education?

- o Yes
- o No
- o Unknown

19. Is there an exam at the end of the

(IF ONE FULL MODULE IS DEDICATED) **module?**

(IF CONTINUOUS PROFESSIONAL EDUCATION) **course?**

- ○ No
- ○ Unknown
- ○ Yes, please specify what type of exam
  ....

20. Is the

(IF ONE FULL MODULE IS DEDICATED) **module also provided at other locations?**

(IF CONTINUOUS PROFESSIONAL EDUCATION) **course provided at multiple locations?**

For example at other universities

- ○ Yes
- ○ No
- ○ Unknown

Q. No. 21 APPEARS ONLY IF “COVERED IN ALL MODULES” WAS SELECTED FOR Q. No. 8.

21. In what way are knowledge and skills with regard to health promotion and disease prevention tested in the curriculum?For example incorporated into a written exam

22. What are the general objectives of the teaching activities on health promotion and disease prevention? e.g. araise awareness among Medical Doctors on the importance of physical activity within the elderly population with a high risk of heart failure

* **23. Is there made use of a mono- or multidisciplinary approach?**

- ○ Monodisciplinary approach
- ○ Multidisciplinary approach
- ○ Unknown

24. The education include theories of:

	1<br>Yes, very much	2<br>Yes, but limited	3<br>No	4<br>Unknown
Population health	☐	☐	☐	☐
Human cognition and behaviour	☐	☐	☐	☐
Health behaviour	☐	☐	☐	☐

*

25. Does the education cover:

	1<br>Yes, very much	2<br>Yes, but limited	3<br>No	4<br>Unknown
Epidemiology and bio-statistics (research capacity)	☐	☐	☐	☐
Methods of evidence-based medicine	☐	☐	☐	☐
Health policies and regulation	☐	☐	☐	☐
Health economics	☐	☐	☐	☐
Ethics	☐	☐	☐	☐
Health care systems	☐	☐	☐	☐
Digitalisation in health promotion	☐	☐	☐	☐
Health inequalities	☐	☐	☐	☐
Disease prevention in teams	☐	☐	☐	☐
Health literacy	☐	☐	☐	☐
Health behaviour change techniques	☐	☐	☐	☐
Communication skills	☐	☐	☐	☐
Digital health coaching	☐	☐	☐	☐

*
26. Concerning teaching methods of health promotion and disease prevention, which methods are used:

	1<br>Yes	2<br>No	3<br>Unknown
Lectures	☐	☐	☐
Assignments	☐	☐	☐
Field training in real environments	☐	☐	☐
eLearning modules	☐	☐	☐
Other, please specify	☐	☐	☐

27. Concerning covered competences or capacities of health promotion and disease prevention, the main focus is on:

*
28. What are the expected outcomes:

	1<br>Yes	2<br>No	3<br>Unknown
Knowledge	☐	☐	☐
Skills	☐	☐	☐
Behaviour/ attitudes	☐	☐	☐

29. Relevant output (if available) e.g. number of students following the module

30. Are there any remarks?Please feel free to give overall remarks or initiative-specific remarks

Your responses have been registered! Thank you for taking the time to complete the survey, your input is valuable to us.

Qs. No. 31-35 APPEAR ONLY FOR CONTINUOUS PROFESSIONAL EDUCATION PROGRAMMES.

31. What are the website details of the education?Please, enter the link of the website with information of the education

32. Can you give a brief description of the education?

33. Which stakeholders were involved in developing the teaching activities on health promotion and disease prevention?

34. Do you think it is likely you will apply the learned knowledge and skills (on health promotion and disease prevention) in daily practice after completion of this education

Yes, please specify

No, please specify

Not sure, please specify

35. Is there a reason why this education might be a good example for other educational settings with regard to the way that health promotion and disease prevention is covered in the education?
