# Supplementary material for: Health promotion and disease prevention in the education of health professionals: a mapping of European educational programmes from 2019
Source: BMC Med Educ. 2022 Nov 11;22:778. doi: 10.1186/s12909-022-03826-5 (PMC9652036; doi:10.1186/s12909-022-03826-5)
Supplement: Supplementary file 2 — Additional file 2. List of countries. This document contains the list of the 31 WHO European Region countries from which educational programmes were included in the mapping. [file 12909_2022_3826_MOESM2_ESM.pdf]

Health promotion and disease prevention in the education of health professionals: a mapping of European educational programmes from 2019

List of the 31 *WHO European Region* countries from which educational programmes were included
(among which 24 were EU MS in 2019)

1. Austria
2. Belgium
3. Bulgaria
4. Cyprus
5. Czech Republic
6. Denmark
7. Estonia
8. Finland
9. France
10. Georgia
11. Greece
12. Hungary
13. Iceland
14. Ireland
15. Italy
16. Kazakhstan
17. Kosovo
18. Latvia
19. Lithuania
20. Malta
21. Netherlands
22. Norway
23. Portugal
24. Romania
25. Serbia
26. Slovakia
27. Slovenia
28. Spain
29. Sweden
30. Switzerland
31. United Kingdom
